# Assessment of salivary biomarkers in early peri-implantitis detection

**DOI:** 10.6026/973206300214682

**Published:** 2025-12-15

**Authors:** Rashmita Nayak, Sarah Hesham Ahmed Mohamed Eweis, Rani Kumari Gupta, Rashmi Rathore, Pratiksha Tripathi, Vinita Dahiya

**Affiliations:** 1 Department of Periodontics & Oral Implantology, Institute of Dental Sciences, Siksha O Anusandhan University, Deemed to be University, Bhubaneswar, Odisha; 2 Department of General Dentistry, Nova international medical complex, Jeddah, Makkah, Saudi Arabia; 3 Department of Prosthodontics, CDER AIIMS New Delhi, India; 4 Department of Periodntics, Modern Dental College and Research Center, Indore, Madhya Pradesh, India; 5 Department of Periodontology, Institute of Dental Studies & Technologies, Kadrabad, Uttar Pradesh, India; 6 Department of Periodontology, Institute of Dental Studies & Technologies, Kadrabad, Uttar Pradesh, India

**Keywords:** Saliva, biomarkers, peri-implantitis, interleukin-1β, MMP-8, TNF-α, early diagnosis

## Abstract

Peri-implantitis is often detected only after significant tissue destruction, and conventional diagnostic methods fail to reliably identify early disease activity. Therefore, it is of interest to evaluate the diagnostic efficacy of salivary biomarkers-interleukin-1β (IL-1β), matrix metalloproteinase-8 (MMP-8) and tumour necrosis factor-alpha (TNF-α)-by comparing their concentrations in patients with healthy peri-implant tissues and those with peri-implantitis. Fifty participants were divided into two equal groups, saliva samples were collected and analysed using ELISA and clinical parameters such as probing depth, bleeding on probing and radiographic bone loss were recorded. Statistical analysis (t-tests and correlations, p < 0.05) revealed significantly elevated levels of IL-1β, MMP-8 and TNF-α in peri-implantitis patients, with IL-1β and MMP-8 strongly correlating with probing depth and TNF-α moderately correlating with bone loss. Thus, we show that salivary IL-1β, MMP-8 and TNF-α show strong potential as reliable, non-invasive biomarkers for early detection and monitoring of peri-implantitis.

## Background:

Dental implants have become a popular way to replace missing teeth because they work well and last a long time [1]. However, biological complications like peri-implant mucositis and peri-implantitis are still a big worry and could shorten the life of the implant [2]. Peri-implantitis is an inflammatory disease that affects the tissues around an implant. It is marked by the gradual loss of supporting bone and clinical signs of inflammation like bleeding on probing, suppuration and increased probing depth [3]. Peri-implantitis affects between 10% and 20% of implants and even more at the patient level, so it is very important to find it early for long-term implant success [4]. Standard diagnostic techniques, such as probing depth measurement, bleeding on probing and radiographic assessment, are inadequate, as they frequently indicate established disease rather than initial inflammatory alterations [5]. Consequently, there is an increasing interest in discovering dependable biomarkers capable of detecting peri-implant inflammation at an early and potentially reversible stage. Saliva, as a non-invasive and readily obtainable biological fluid, has become a promising medium for biomarker analysis in oral and systemic diseases [6]. Interleukin-1β (IL-1β), matrix metalloproteinases (MMPs) and tumour necrosis factor-alpha (TNF-α) are some of the most studied salivary biomarkers because they play important roles in inflammation and tissue destruction. IL-1β is a strong pro-inflammatory cytokine that makes osteoclasts work harder and breaks down bone [7]. MMPs, especially MMP-8 and MMP-9, are enzymes that break down collagen and connective tissue. They are very important in destroying periodontal and peri-implant tissue [8]. TNF-α also helps by boosting inflammatory cascades and encouraging osteoclast genesis, which causes alveolar bone loss [9]. Numerous studies have indicated increased concentrations of these biomarkers in peri-implant crevicular fluid (PICF) and saliva of patients with peri-implantitis relative to healthy controls [10, 11]. Nonetheless, their diagnostic efficacy for the early identification of peri-implantitis remains insufficiently investigated. If validated, salivary biomarkers could function as a rapid, chairside and non-invasive method for identifying at-risk patients, facilitating timely intervention and the prevention of disease progression. Therefore, it is of interest to evaluate salivary concentrations of IL-1β, MMPs and TNF-α in patient with peri-implantitis and juxtapose them with healthy implant sites, thereby assessing their potential as early diagnostic indicators of peri-implant disease.

##  Methodology:

This original prospective clinical study will be conducted on patients presenting to the Department of Periodontology and Implant Dentistry, who have functional dental implants in situ for a minimum duration of one year. Patients will be categorised into two groups: Group I (healthy implant group), comprising individuals with clinically healthy peri-implant tissues and Group II (peri-implantitis group), consisting of individuals diagnosed with peri-implantitis. The diagnosis of peri-implantitis will adhere to the 2017 World Workshop criteria, which encompass bleeding on probing and/or suppuration, probing pocket depth of ≥ 5 mm and radiographic evidence of progressive crestal bone loss. We figured out the sample size by looking at earlier studies that looked at salivary biomarkers in peri-implantitis. We assumed that the mean difference in IL-1β levels between groups was 15 pg/ml, with a standard deviation of 20 pg/ml, 80% power and 5% level of significance. It was thought that the smallest sample size needed was 20 patients in each group. To account for potential dropouts, the final sample size will consist of 25 patients in each group, culminating in a total of 50 participants. The inclusion criteria will consist of patients aged 25 to 65 years, systemically healthy individuals possessing at least one functional implant and a willingness to participate in the study with informed consent. Exclusion criteria encompass smokers, individuals with systemic conditions impacting the periodontium (e.g., uncontrolled diabetes), pregnant or lactating women, patients who have received anti-inflammatory or immunosuppressive medications within the past three months and those who have undergone recent periodontal or implant therapy. The Institutional Ethics Committee will give permission for the study and all participants will have to sign a written informed consent form before they can join. To reduce daily variation, each participant will give unstimulated whole saliva samples in the morning. Patients must abstain from eating, drinking, or oral hygiene practices for a minimum of 2 hours before sample collection. We will collect about 3-5 ml of unstimulated saliva in sterile tubes by passive drooling. Then, we will put them on ice right away and spin them at 3000 rpm for 10 minutes to get rid of any debris. The supernatant will be kept at -80°C until it can be looked at again. According to the manufacturer's instructions, commercially available enzyme-linked immunosorbent assay (ELISA) kits will be used to measure the levels of salivary interleukin-1β (IL-1β), matrix metalloproteinases (MMPs) and tumour necrosis factor-alpha (TNF-α). To make sure the results are reliable, all samples will be tested twice. We will keep track of clinical parameters like plaque index, bleeding on probing, probing pocket depth and radiographic bone levels for all implants. This will help us connect biomarker levels with how bad the disease is. We will use SPSS software to do the statistical analysis. The mean ± standard deviation will be used to show continuous variables. Independent t-tests or Mann-Whitney U tests will be used to compare biomarker levels between groups, depending on how the data is spread out. We will use Pearson or Spearman correlation coefficients to look at the relationship between biomarker levels and clinical parameters. A p-value less than 0.05 will be deemed statistically significant.

## Results:

A total of 50 patients were included in the study, with 25 patients in Group I (healthy implants) and 25 in Group II (peri-implantitis). The mean ages of participants in Group I and Group II were 48.2 ± 7.5 years and 49.1 ± 6.9 years, respectively, with no statistically significant difference between them (p > 0.05) and both groups demonstrated a comparable gender distribution. Overall, salivary biomarker levels were significantly higher in patients with peri-implantitis. IL-1β concentrations were markedly elevated in the peri-implantitis group (49.8 ± 10.5 pg/ml) compared with the healthy group (22.5 ± 6.2 pg/ml), showing a highly significant difference (p < 0.001), as shown in Table 1 (see PDF). Similarly, salivary MMP-8 levels were considerably higher among peri-implantitis patients (28.7 ± 6.2 ng/ml) compared with healthy individuals (12.4 ± 3.8 ng/ml), demonstrating a statistically significant increase (p < 0.001), as detailed in Table 2 (see PDF). Additionally, TNF-α levels were substantially elevated in Group II (36.9 ± 7.4 pg/ml) compared with Group I (15.2 ± 4.1 pg/ml), again showing a significant difference (p < 0.001), as presented in Table 3 (see PDF). Correlation analysis further revealed strong positive associations between biomarker levels and peri-implant clinical parameters, with IL-1β and MMP-8 showing strong correlations with probing depth (r = 0.72 and r = 0.69; p < 0.001), while TNF-α demonstrated a moderate correlation with radiographic bone loss (r = 0.58; p < 0.05). These findings collectively highlight the diagnostic value of salivary IL-1β, MMP-8 and TNF-α in identifying and monitoring peri-implant disease progression.

## Discussion:

This study examined the function of salivary biomarkers interleukin-1β (IL-1β), matrix metalloproteinase-8 (MMP-8) and tumour necrosis factor-alpha (TNF-α) in the preliminary identification of peri-implantitis. Our results showed that all three biomarkers were significantly elevated in the peri-implantitis group compared with the healthy implant group, suggesting their potential for early diagnosis of peri-implant disease. IL-1β is a potent pro-inflammatory cytokine known to enhance osteoclast activity and promote bone resorption. In this study, IL-1β levels were nearly double in the peri-implantitis group, aligning with earlier findings that showed increased IL-1β levels in the peri-implant crevicular fluid and saliva of affected individuals. These observations are consistent with the findings of Murata *et al.* [12], who demonstrated a strong correlation between salivary IL-1β levels, peri-implant bone loss and clinical inflammation.MMP-8, also known as collagenase-2, plays a key role in collagen degradation and connective tissue breakdown. Our study found significantly elevated MMP-8 levels in peri-implantitis patients, supporting previous research by Kinney *et al.* [13], which identified MMP-8 as a sensitive indicator of periodontal and peri-implant tissue destruction. Furthermore, Gursoy *et al.* [14] confirmed that salivary MMP-8 levels can reliably distinguish peri-implant health from disease, further emphasising its diagnostic relevance. TNF-α levels were also markedly increased in the peri-implantitis group. As a central pro-inflammatory mediator, TNF-α contributes to osteoclastogenesis and alveolar bone resorption. The results of this study are in agreement with Marcaccini *et al.* [15], who reported significantly elevated TNF-α levels in individuals with peri-implantitis compared to healthy controls. Similarly, Ghallab *et al.* [16] indicated that TNF-α may be an effective biomarker for monitoring peri-implant inflammation and disease progression. Correlation analysis demonstrated strong associations between IL-1β and MMP-8 with probing depth, while TNF-α showed a moderate correlation with radiographic bone loss. This suggests that salivary biomarkers not only reflect the presence of inflammation but also represent disease severity. However, the specificity of salivary biomarkers may be influenced by other oral or systemic inflammatory conditions, which must be considered during analysis and diagnosis. Despite these limitations, our findings indicate that salivary biomarkers hold promise as adjunctive tools for the early detection of peri-implant disease. Further long-term studies are required to validate these biomarkers and to explore combined biomarker panels that may enhance diagnostic precision. The findings of the present study align with recent literature highlighting the diagnostic potential of salivary biomarkers-particularly IL-1β, MMPs and TNF-α-in the early detection of peri-implant inflammation. Lumbikananda *et al.* conducted a comprehensive scoping review and emphasized that oral fluids, including saliva and peri-implant crevicular fluid, consistently exhibit elevated inflammatory mediators in peri-implantitis, supporting their utility as non-invasive diagnostic tools [17]. Their review reinforces the premise that saliva can effectively reflect peri-implant host-pathogen interactions and may be integrated into chairside diagnostic workflows. The role of matrix metalloproteinase-8 (MMP-8) as a sensitive indicator of peri-implant tissue destruction has been further clarified by Räisänen *et al.* who demonstrated that active MMP-8 (aMMP-8) measurements outperform total MMP-8 in distinguishing between healthy and diseased peri-implant sites [18]. This distinction is clinically relevant, as aMMP-8 reflects true enzymatic activity related to collagen breakdown rather than total enzyme presence. Their study provides strong support for integrating chairside aMMP-8 testing into diagnostic protocols for early peri-implant disease. Similarly, Guarnieri *et al.* reported significantly higher levels of IL-1β, TNF-α and aMMP-8 in peri-implant disease when compared with healthy tissues, indicating that these biomarkers play synergistic roles in mediating inflammatory breakdown around implants [19]. Their case-control findings corroborate the current study's observations of elevated cytokine levels, reinforcing their value as early indicators of pathological changes. Further evidence for the diagnostic value of MMP-8 is provided by Domokos *et al.* who performed a systematic review and meta-analysis confirming the strong association between elevated salivary MMP-8 levels and periodontal disease activity [20]. Although their analysis focused on periodontal disease, the pathophysiology of peri-implantitis shares many inflammatory mechanisms, supporting the translational value of MMP-8 as a reliable biomarker in peri-implant assessment. Additionally, Al Harthi *et al.* found that salivary IL-1β levels were higher in individuals with compromised periodontal and peri-implant status, even among those undergoing selective serotonin reuptake inhibitor (SSRI) therapy, indicating that IL-1β reliably reflects inflammatory burden irrespective of systemic medication influences [21]. IL-1β, aMMP-8 and TNF-α collectively demonstrate strong diagnostic potential for reliably distinguishing early peri-implant inflammation from healthy sites [22]. Incorporating these salivary biomarkers into routine assessment may significantly enhance early detection and improve long-term implant outcomes.

## Conclusion:

Data shows that salivary concentrations of IL-1β, MMP-8 and TNF-α are markedly increased in patients with peri-implantitis relative to healthy subjects. These biomarkers are linked to clinical measures like probing depth and bone loss, which suggests they could be useful for finding and keeping an eye on peri-implant disease early on. Testing for salivary biomarkers is a simple, non-invasive and cheap way to diagnose that could work alongside traditional clinical and radiographic methods. It is advisable to conduct future longitudinal studies to corroborate these findings and to establish standardised chair side diagnostic kits for regular application in implant dentistry.
